# Synchronous association of rectal adenocarcinoma and three ileal carcinoids: a case report

**DOI:** 10.1186/1477-7819-7-21

**Published:** 2009-02-19

**Authors:** Seamus M McHugh, Jill O'Donnell, Peter Gillen

**Affiliations:** 1 Department of Surgery, Our Lady of Lourdes Hospital, Drogheda, Ireland, UK

## Abstract

**Background:**

Synchronous midgut carcinoids with gastrointestinal adenocarcinoma are a rare but recognised association.

**Case presentation:**

The patient, a 74 year old woman, underwent anterior resection for a low rectal adenocarcinoma. Intra-operatively 3 serosal deposits of tumour were noted in the distal ileum. Histology revealed these to be ileal carcinoids.

**Conclusion:**

During resection of a gastrointestinal tumour, a thorough inspection of the abdominal cavity should be undertaken to investigate the possibility of metastatic secondaries or a synchronous tumour as is reported in this case.

## Background

The natural history of midgut carcinoid tumours is to progress slowly, arising from neuro-endocrine cells that line the tract. They often present with metastasis at diagnosis and occur most frequently in the ileum (52%) and the appendix (22%) [1]. The incidence of ileal carcinoids appears to be increasing [2]. The concept of an association with a synchronously occurring non-carcinoid neoplasia was first broached in 1949 [3], and several reviews since have stressed this connection [4-7]. We present the case of a 74 year old woman who underwent elective anterior resection for a high rectal adenocarcinoma with the incidental discovery of 3 ileal carcinoids during her surgery.

## Case presentation

**Figure 1:**
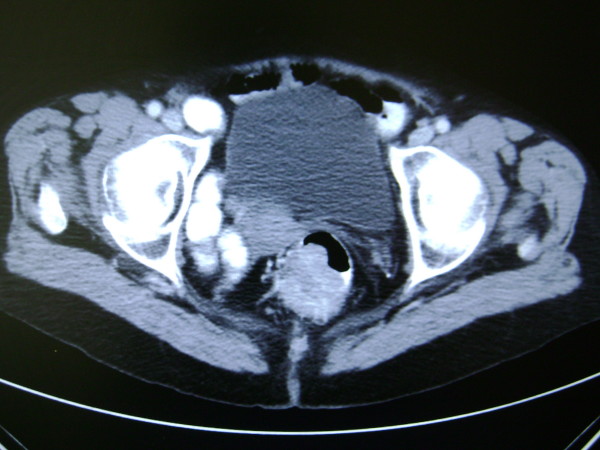
**Pre-operative CT Abdomen-Pelvis image showing polypoid rectal lesion extending into the lumen**.

**Figure 2:**
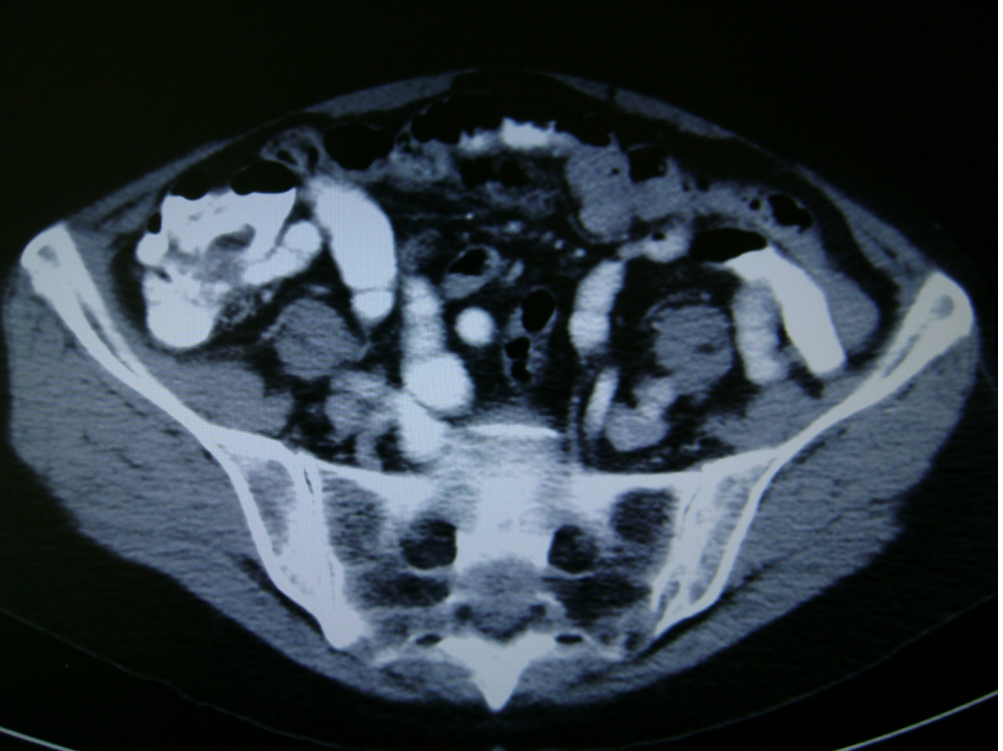
**Pre-operative CT Abdomen Pelvis image showing normal appearing ileum at ileo-caecal junction**.

The patient was referred to by her G.P. to surgical outpatients with a 9 month history of diarrhoea with tenesmus. Physical exam was normal, including a soft non-tender abdomen. Serum full blood count, urea & electrolytes, liver function tests and coagulation screen were all within normal limits. Elective colonoscopy revealed a large villous tumour in the lower rectum, which proved to be a moderately differentiated adenocarcinoma histologically. Staging abdomino-pelvic CT & MRI scans confirmed a 6.5 × 4 cm T3N1 irregular low rectal mass lesion extending inferiorly with involvement of the muscularis and serosa (figure 1), but no distal metastasis or small bowel pathology (figure 2).

Neo adjuvant chemo-radiotherapy was given to downstage the tumour and repeat MRI scan showed marked reduction of the mass lesion with no evidence of lymphadenopathy.

At laparotomy a mobile tumour was noted in the upper rectum following mobilisation. In addition three serosal deposits of tumour were noted in the distal ileum. An anterior resection was performed and 25 cm of ileum was also resected

The patient made an uncomplicated recovery and was discharged on 15 post operative day.

Two nodules of carcinoid were confirmed in the small bowel segment using immuno-staining. They both invaded to serosal level. In the third, tumour was lost on deeper sectioning but it's H&E appearance supported carcinoid. Two tiny mesenteric nodes taken were clear. The 12 cm segment of rectum showed radiation change but no residual primary mucosal lesion, demonstrating complete pathological response with 12 negative lymph nodes.

## Discussion

In 1888, Otto Lubarsch, a pathologist based in Berlin first described carcinoid lesions in detail. He reported the autopsy findings of a patient with multiple carcinoid tumours involving the ileum. Carcinoid syndrome and it's classic associated symptoms was reported two years later by Ransom. He described a patient with diarrhoea and wheezing secondary to an ileal carcinoid which had a distant metastasis to the liver [8].

The annual age-adjusted incidence of small intestine carcinoids is reported as increasing, from 1.09 per 100,000 in 1973 to 5.25 per 100,000 in 2004 [9]. The incidental finding of malignant carcinoid at autopsy is reported at 21 per million [10]. Surgical resection is the preferred treatment option. With regards neoadjuvant therapy, combined associations (including either 5-fluorouracil and/or streptozotocin) rarely exceed a 20% response rate [11].

Five year survival for resected isolated carcinoid is determined by site, with appendiceal carcinoids having a better 5 year survival prognosis (> 95%) than small intestine carcinoids (70–80%) [12]. Recent European guidelines for surveillance of midgut carcinoids post resection with curative intent suggest follow up every 6–12 months, with the exception of grade 3 tumours which should be followed every 3 months. Minimal examinations include measurement of chromogranin A (a neuroendocrine secretory protein located in the secretory vesicles of neurons and neuroendocrine cells) and 5-Hydroxyindoleacetic acid (5-HIAA is the main metabolite of serotonin) in 24 hour urine and with three-phasic CT scan [13]. Follow up should be life-long.

Synchronous carcinoids with non-carcinoid neoplasms in the G.I. tract were first noted by Pearson and Fitzgerald in 1949 [2]. A study by Gerstle et al in 1995 reported on 69 patients with carcinoids of the gastrointestinal tract were discovered, of whom 29 (42 percent) had second synchronous tumours [14]. To our knowledge this is the first report with as many as 3 midgut carcinoids discovered with a synchronous adenocarcinoma. This further cements the association elucidated by Gerstle et al.

Hypotheses put forward to explain this association include the secretion of active neuroendocrine peptides such as gastrin and cholecystokinin [15]. Both have been previously implicated as directly regulating growth in colorectal carcinoma [16]. Non-neuroendocrine peptides regulating cell growth and differentiation have been demonstrated in gastrointestinal carcinoid tumours and may also play a role in carcinogenesis [17].

## Conclusion

In the case of a synchronous carcinoid with adenocarcinoma, management is directed towards the carcinoma, since the finding of carcinoid is incidental and so it is usually at an early stage. During resection of the colorectal tumour, a thorough inspection of the abdominal cavity should be undertaken to investigate the possibility of metastatic secondaries or a synchronous tumour as is reported in this case. Because of their slow growing natural history, the discovery of an asymptomatic gastrointestinal carcinoid during the operative treatment of another malignancy usually requires resection alone without additional treatment and will have little effect on the prognosis of the individual [18].

## Consent

Written consent was obtained from the patient for publication of this case report

## Competing interests

The authors declare that they have no competing interests.

## Authors' contributions

SM was involved in data acquisition and interpretation, writing initial drafts and subsequent revisions and undertook review of literature on topic. JOD made a substantial contribution regarding conception of report, was the editor of multiple drafts of case report, made many suggestions regarding format of report for inclusion of intellectual content and undertook review of literature on topic. PG made a substantial contribution regarding concept of case report, was the supervisor of work done and drafts of case report prepared by first and second authors. All authors read and approved final approval of version to be published given.
